# DGG-100629 inhibits lung cancer growth by suppressing the NFATc1/DDIAS/STAT3 pathway

**DOI:** 10.1038/s12276-021-00601-2

**Published:** 2021-04-15

**Authors:** Joo-Young Im, Bo-Kyung Kim, Sung-Hoon Yoon, Byoung Chul Cho, Yu Mi Baek, Mi-Jung Kang, Nayeon Kim, Young-Dae Gong, Misun Won

**Affiliations:** 1 Personalized Genomic Medicine Research Center, KRIBB, Daejeon, 34141 Korea; 2 National Center for Efficacy Evaluation for Respiratory Disease Product, Korea Institute of Toxicology, Jeongeup, Jeollabuk-do 56212 Korea; 3 Department of Human and Environmental Toxicology, University of Science and Technology (UST), Daejeon, 34113 Korea; 4 Division of Medical Oncology, Yonsei University College of Medicine, Seoul, 03722 Korea; 5 Therna Therapeutics, Yangcheon-ro, Gangseo-gu, Seoul 05029 Korea; 6 Innovative Drug Library Research Center, Department of Chemistry, College of Science, Dongguk University, Seoul, 04620 Korea; 7 Deparment of Functional Genomics, University of Science and Technology (UST), Daejeon, 34113 Korea

**Keywords:** Reporter genes, Non-small-cell lung cancer, Apoptosis, Targeted therapies, Drug development

## Abstract

DNA damage-induced apoptosis suppressor (DDIAS) promotes the progression of lung cancer and hepatocellular carcinoma through the regulation of multiple pathways. We screened a chemical library for anticancer agent(s) capable of inhibiting DDIAS transcription. DGG-100629 was found to suppress lung cancer cell growth through the inhibition of DDIAS expression. DGG-100629 induced c-Jun NH(2)-terminal kinase (JNK) activation and inhibited NFATc1 nuclear translocation. Treatment with SP600125 (a JNK inhibitor) or knockdown of JNK1 restored DDIAS expression and reversed DGG-100629-induced cell death. In addition, DGG-100629 suppressed the signal transducer and activator of transcription (STAT3) signaling pathway. DDIAS or STAT3 overexpression restored lung cancer cell growth in the presence of DGG-100629. In a xenograft assay, DGG-100629 inhibited tumor growth by reducing the level of phosphorylated STAT3 and the expression of STAT3 target genes. Moreover, DGG-100629 inhibited the growth of lung cancer patient-derived gefitinib-resistant cells expressing NFATc1 and DDIAS. Our findings emphasize the potential of DDIAS blockade as a therapeutic approach and suggest a novel strategy for the treatment of gefitinib-resistant lung cancer.

## Lung cancer: Treating tumors resistant to standard therapy

A drug targeting a protein implicated in cancer cell survival could offer a new way of treating lung tumors that are resistant to standard therapy. A South Korean team led by Misun Won, Korea Research Institute of Bioscience & Biotechnology, Daejeon, and Young-Dae Gong, Dongguk University, Seoul, screened a library of 1,500 chemicals looking for compounds that suppressed the activity of DDIAS, a protein preventing programmed cell death. Their search yielded a molecule that blocked a transcription factor from entering the nucleus and inducing the expression of DDIAS. In mice with implanted lung tumors, treatment with this molecule inhibited cancer growth and blocked downstream signaling of DDIAS. It also blunted the proliferation of cancer cells taken from patients who were resistant to gefitinib, a targeted drug therapy often used to treat lung tumors.

## Introduction

Lung cancer is a leading cause of cancer mortality worldwide and constitutes a heterogeneous group of tumors^1^. Non-small cell lung cancer (NSCLC) accounts for 80–85% of all lung cancers and is the most prevalent subtype^2^. Current treatments include surgery, platinum-based chemotherapy, targeted therapy, and immunotherapy. However, the prognosis is poor, and the 5-year survival rate is less than 20%^2^.

DNA damage-induced apoptosis suppressor (DDIAS) is aberrantly expressed in human lung cancer, colon cancer, and hepatocellular carcinoma^3,4^. The level of DDIAS expression is correlated with clinical progression and predicts poor survival in patients with hepatocellular carcinoma and lung cancer^3,5^. DDIAS is involved in the resistance of lung cancer to DNA-damaging agents, such as cisplatin and camptothecin, as well as in the resistance of lung cancer and hepatocellular carcinoma to tumor necrosis factor-related apoptosis-inducing ligand (TRAIL)^3,4,6,7^. DDIAS is induced by nuclear factor of activated T cells (NFATc)1 and myocyte enhancer-binding factor 2B (MEF2B) and is posttranslationally regulated by the E3 U-box ubiquitin ligase CHIP^6,8,9^. In addition, DDIAS promotes cell proliferation through the p38-ATF2 pathway in breast cancer cells and interacts with DNA polymerase, promoting tumorigenesis in hepatocellular carcinoma^3,10^. Recently, we reported that DDIAS promotes STAT3 signaling by directly binding to STAT3, thus preventing its interaction with PTPRM and causing increased STAT3 phosphorylation^11^. Moreover, we determined that miconazole inhibits the DDIAS/STAT3 interaction, resulting in anticancer activity in lung cancer^12^. Therefore, DDIAS may be a therapeutic target, and specific DDIAS-targeting agents should be developed.

Quinoxalines are well-known heterocyclic compounds bearing a benzene ring and a pyrazine ring. Diversely substituted quinoxalines and their derivatives exhibit important therapeutic activities as anticancer, anti-inflammatory, antimicrobial, and antidepressant agents. A novel series of ethyl 3-(arylethynyl)quinoxaline-2-carboxylates, indeno[1,2-b] quinoxaline derivatives, sulfonamide quinoxaline, and quinoxaline-derived chalcones exhibit anticancer activity^13–16^. In addition, 2,3,6-trisubstituted quinoxaline derivatives and 3-arylethynyl-substituted thieno[3,4-b]-pyrazine derivatives were identified as Wnt2/β-catenin pathway inhibitors and transglutaminase 2 inhibitors in NSCLC and renal cancer cells, respectively^17,18^.

Previously, we demonstrated that the transcription of *DDIAS* is regulated by binding of NFATc1 to the *DDIAS* promoter. In this study, a reporter assay based on DDIAS promoter-controlled transcription was used to screen a chemical library for DDIAS-targeting anticancer agents. We report that the quinoxaline derivative DGG-100629 was able to suppress DDIAS transcription. In particular, DGG-100629 suppressed the nuclear translocation of NFATc1 through JNK activation, resulting in inhibition of DDIAS expression and STAT3 signaling. These findings suggest that NFATc1 functionally links the immune system to DDIAS and STAT3-mediated oncogenic signaling, which in turn promotes the proliferation and survival of lung cancer cells.

## Materials and methods

### Reagents and plasmids

An innovative drug library (1500 compounds) was provided by Dongguk University as 5 mM stock solutions. DGG-100629, DGG-100647, and DGG-108632, synthesized by Prof. Gong, were suspended in dimethyl sulfoxide. Sulforhodamine B was obtained from Sigma-Aldrich (St. Louis, MO, USA). SP600125 and CHIR99021 were purchased from Selleckchem (Houston, TX, USA). The following antibodies were used: anti-DDIAS (HPA038540) from Atlas Antibodies (Stockholm, Sweden); anti-NFATc1 (sc-7294), anti-NFATc2 (sc-7295), anti-NFATc4 (13036), anti-PARP (7150), anti-HDAC1 (sc-6298), anti-GFP (sc-9996), and anti-HA (sc-805) from Santa Cruz Biotechnology (Dallas, TX, USA); anti-STAT3 (#9139, #4904), anti-NFATc3 (#4998), anti-p-JNK (#9251), anti-JNK (#9252), anti-pGSK3β (#9336), anti-GSK3β (#9315), anti-HA (#3724), anti-Bcl-xL (#2762), anti-pSTAT3 (Y705, #9145), anti-Caspase-3 (#9662), and anti-Survivin (#2803) from Cell Signaling Technology (Beverly, MA, USA); anti-GAPDH (LF-P-A0212) from AbFrontier (Seoul, Korea); and anti-Flag (F1804) from Sigma-Aldrich.

Flag-DDIAS and STAT3-tagged hemagglutinin (HA) and the P3 and P5 fragments of the DDIAS promoter were previously described^6,11^. The P3 fragment of the DDIAS promoter (pGL4-P3) was subcloned into pGL4.17, which contained the sequence of a neomycin resistance gene as a selection marker (Promega, Madison, WI, USA). GFP-NFATc1 (#24219) was obtained from Addgene (Cambridge, MA, USA). The constructs were confirmed by sequence analysis.

### Cell culture and transfection

Human cervical cancer HeLa cells, as well as non-small cell lung cancer NCI-H23, NCI-H1703, NCI-H358, and Calu-3 cells, were purchased from the Korean Cell Line Bank (Seoul, Korea) or KRIBB Cell Line Bank (Daejeon, Korea). The lung cancer cell lines YL01, YL03, YL05 (EGFR exon19del), and YL08 (EGFR wild-type/ALK mutation-positive), each derived from a different patient, were provided by the Yonsei University College of Medicine, Seoul, Korea. Patient characteristics and treatments were previously described^19^. Cells were cultured in Dulbecco’s modified Eagle’s medium (DMEM) or RPMI-1640 medium supplemented with 10% fetal bovine serum (FBS) and penicillin/streptomycin (Invitrogen, Carlsbad, CA, USA). Cells were transfected with plasmids using Turbofect (Thermo Scientific, Rockford, IL, USA) and with siRNAs (20–40 nM) by electroporation (Neon, Invitrogen) according to the manufacturer’s instructions. The siRNAs used in this study were purchased from Bioneer Corporation (Daejeon, Korea). The target sequences were as follows: siJNK1 #1, 5′-CUGGUAUGAUCCUUCUGAA-3′; siJNK1 #2, 5′-GUCACACCUGGAAACCUGA-3′; and siScrambled, 5′-CCUACGCCACCAAUUUCGU-3′.

### Establishment of a stable cell line containing the human *DDIAS* promoter

HeLa cells were transfected with the P3 fragment (pGL4-P3) of the *DDIAS* promoter and selected with 1 mg/mL G418 for 10 days. The selected clones were picked and cultured with 1 mg/mL G418. Each clone was analyzed by a luciferase reporter assay, and one clone was used in this study.

### Cell growth assay and IncuCyte system

We performed a cell growth inhibition assay by treating cells (3000 cells/96-well plate) for 72 h with DGG-100629 and calculated the GI50 (half-maximal cell growth inhibitory concentration). Cell growth was determined by using a sulforhodamine B assay, as previously described^20^. Cell death was analyzed with CellPlayer reagent-based annexin V staining according to the manufacturer’s protocols (Essen Bioscience, Ann Arbor, MI, USA). Green or red fluorescence and phase contrast images were acquired using a ×10 objective.

### Luciferase reporter assay

Stable HeLa cells were treated with the compounds for 9 h and lysed with 1× passive lysis buffer in 48-well plates. For transient transfection, HeLa cells were cotransfected with 300 ng of pGL2, pGL2-P3, or pGL2-P5 and 10 ng of pRL-TK (thymidine kinase promoter-Renilla luciferase reporter plasmid) by using 2 μL of Turbofect (Thermo Scientific) per well. Twenty-four hours after transfection, the cells were treated with the compounds for 9 h and lysed. Firefly and Renilla luciferase activities were evaluated using a dual-luciferase reporter assay system (Promega) and a luminometer (Victor X light, Perkin Elmer, Waltham, MA, USA). Firefly luciferase activity was normalized to Renilla luciferase activity and expressed as relative luciferase activity (RLA), indicating the level of promoter transcription.

### Reverse transcription-polymerase chain reaction

Two micrograms of total RNA was isolated using TRIzol (Invitrogen) and reverse transcribed into cDNA by using a TOPscript RT kit (Enzynomics, Daejeon, Korea) according to the manufacturer’s protocol. The primers used were as follows: DDIAS F, 5′-CTTGCAGCAGTTGTTACGAA-3′ and R, 5′-GTGACCAAGCACTTCGAGTT-3′; GAPDH: 5′-CCUACGCCACCAAUUUCGU-3′. All reactions were performed in triplicate and normalized to the level of *GAPDH* as the internal control.

### Immunoblot analysis

DDIAS protein expression was examined at 24 h (2 × 10^5^ cells/6-well plate) after DGG-100629 treatment. Cells were lysed with 0.5% NP40 or 1× RIPA buffer (Millipore, Temecula, CA, USA) containing 1 mM Na_3_VO_4_, 1 mM sodium fluoride, 1 mM PMSF, and protease inhibitor cocktail (Roche, Basel, Switzerland). Protein concentrations in the lysates were quantified using a BCA assay kit (Bio-Rad, Hercules, CA, USA). Nuclear extracts were prepared as previously described^21^. The lysates were subjected to immunoblotting with specific antibodies. Western blot band signals were detected with an enhanced chemiluminescence (ECL) kit (Millipore).

### Chromatin immunoprecipitation assay

ChIP assays were performed by using an EZ-ChIP kit (Millipore, Billerica, MA, USA) according to the manufacturer’s instructions. Chromatin was sheared by sonication on ice. The chromatin solution was incubated with 2 μg of an anti-NFATc1 antibody or normal mouse immunoglobulin G (IgG; negative control; Santa Cruz Biotechnology) overnight at 4 °C. These isolated DNA fragments were used as templates for PCR analysis. The following primers were used to amplify the NFAT consensus binding sites located in the *DDIAS* promoter region, as previously described^6^: forward for a, 5′-TGCTAGCCCCTAGGACAGCGC-3′ and reverse, 5′-TCCCCGACAGGTGGATCAGTA-3′; forward for b, 5′-TACGCCCAGGAGGCTCAGCGAA-3′ and reverse, 5′-CCGCGTCCTTTTCCGCCGGAA-3′. All ChIP experiments were performed at least twice.

### Mouse xenograft assay

All mouse studies were conducted in accordance with a protocol approved by the Institutional Animal Care and Use Committee. Tumors were established by injecting NCI-H1703 cells (9 × 10^6^ cells/mouse) subcutaneously into 6-week-old BALB/c female nude mice (Nara Biotech., South Korea). Tumor volumes were estimated by the following formula: length (mm) × width (mm) × height (mm)/2. When the average tumor volume was 57.5 cm^3^, the mice were randomly divided into two groups (*n* = 5 mice/group), and DGG-100629 (10 mg/kg) was administered intraperitoneally once a day for 18 days. The mice were euthanized on day 18, and the tumor weights were measured.

### Statistical analysis

All data were obtained from at least three independent experiments. The results are expressed as the mean ± S.E.M. values. Statistical analyses were performed by using a two-tailed Student’s *t*-test. *P* values < 0.05 were considered significant.

## Results

### Screening and identification of inhibitors of DDIAS expression

**Fig. 1:**
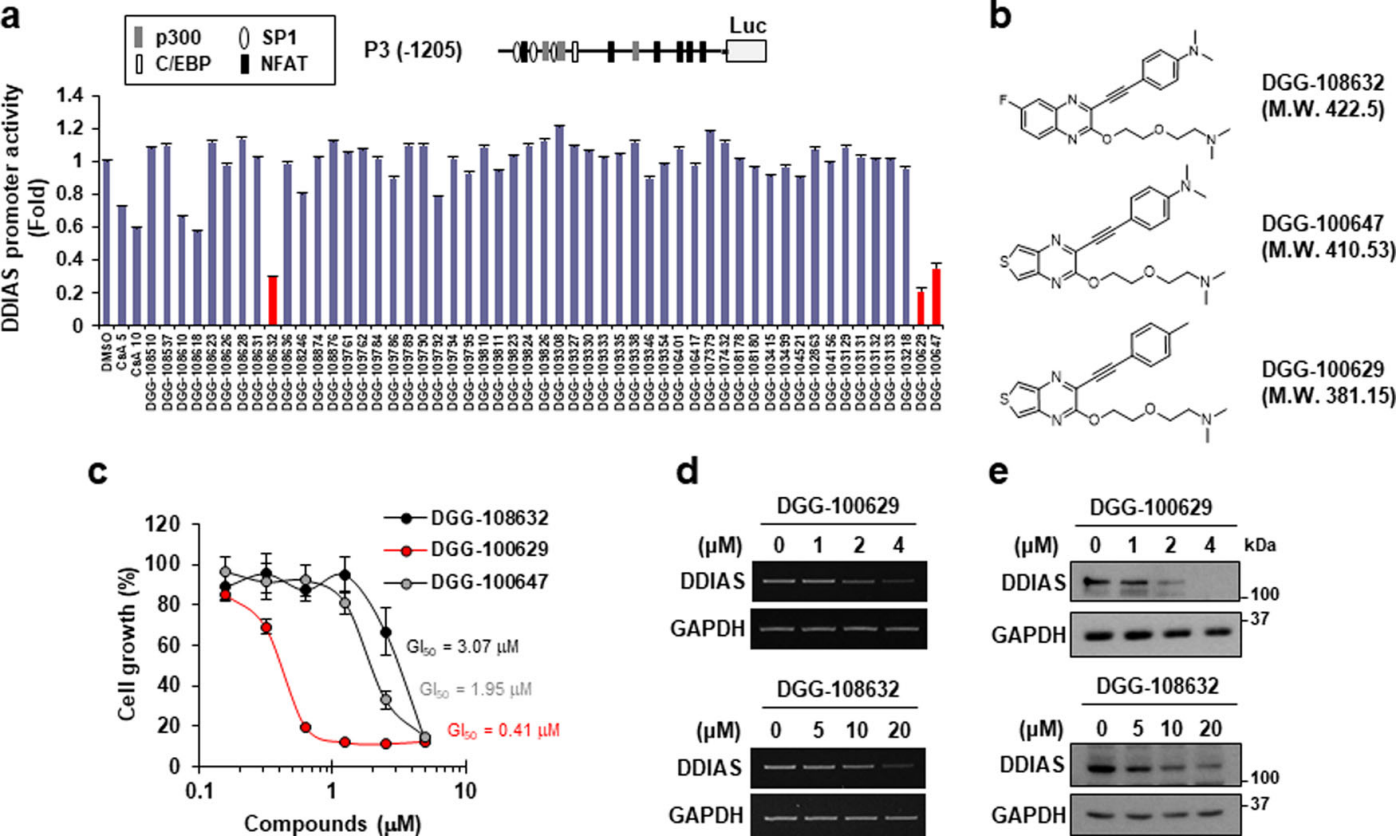
Screening of suppressors of DDIAS expression. **a** HeLa-pGL4-P3 cells were treated with the compounds (5 μM) for 9 h. Cell lysates were analyzed by luciferase reporter assays. Cyclosporine A (CsA) was used as the positive control. The DDIAS promoter P3 fragment was located 1205 bp upstream of the luciferase gene (upper panel). **b** Structures of three selected compounds. **c** HeLa cells were treated with the compounds for 72 h. Cell growth was evaluated by a sulforhodamine B (SRB) assay. **d** Assessment of DDIAS mRNA levels by quantitative RT-PCR in HeLa cells treated for 12 h with the compounds. **e** Western blot analysis of DDIAS protein levels in HeLa cells treated with the compounds for 24 h.

To search for drugs capable of inhibiting DDIAS expression, HeLa cells (HeLa-pGL4-P3) stably expressing the luciferase reporter gene under the control of the DDIAS promoter (P3, −1205/+125) were established (Fig. 1a). A total of 1500 compounds from an innovative drug library provided by Dongguk University were screened for their ability to inhibit HeLa-pGL4-P3 cell growth. Of these compounds, 53 that suppressed the growth of HeLa-pGL4-P3 cells by more than 50% at 5 μM but did not affect the growth of WI-38 normal human lung fibroblasts were selected (data not shown). Then, the effect of these 53 compounds on DDIAS promoter activity was examined. Notably, three compounds, DGG-108632, DGG-100629, and DGG-100647, inhibited DDIAS promoter activity by more than 60% at 5 μM (Fig. 1a). The three compounds, which shared a 2,3,4-trisubstituted quinoxaline scaffold (Fig. 1b), were more effective at inhibiting DDIAS promoter activity than cyclosporine A (CsA), an inhibitor of calcineurin that also suppresses DDIAS expression^6^. Next, the effect of DGG-108632, DGG-100629, and DGG-100647 on cell growth was examined. DGG-100629 displayed a lower GI50 value (GI_50_ = 0.41 μM) than both DGG-108632 (GI_50_ = 3.07 μM) and DGG-100647 (GI_50_ = 1.95 μM) (Fig. 1c) and did not affect the growth of WI-38 cells (Supplementary Fig. 1). DGG-108632 and DGG-100629 decreased the mRNA and protein levels of DDIAS in a dose-dependent manner (Fig. 1d, e). These data suggested that DGG-100629 suppressed the growth of cancer cells by inhibiting DDIAS transcription.

### NFATc1 was required for DGG-100629-induced suppression of DDIAS expression

**Fig. 2:**
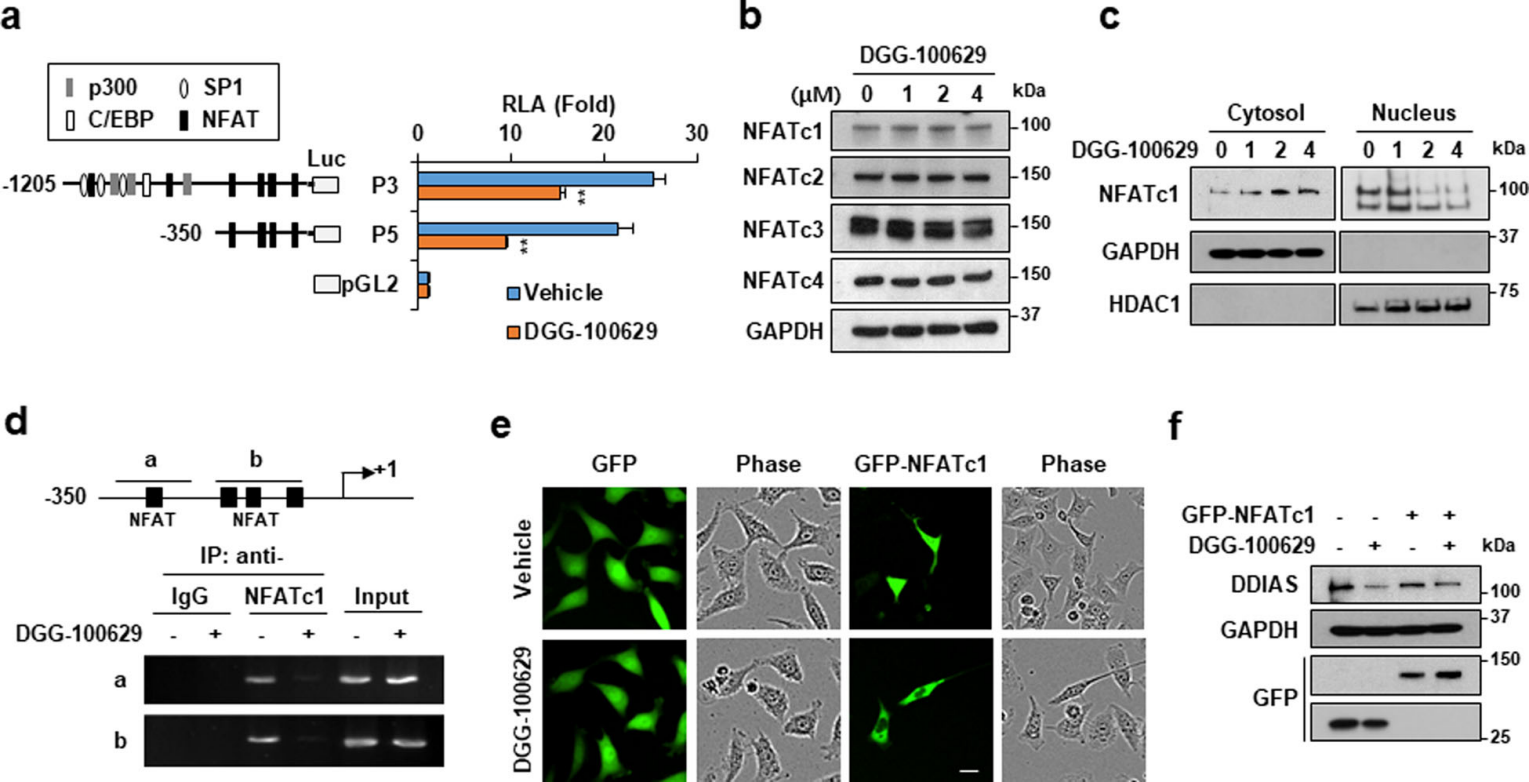
DGG-100629 inhibited DDIAS expression. **a** HeLa cells were cotransfected with DDIAS promoter-containing luciferase reporter plasmids (P3, P5, pGL2) and control plasmids (pRL-TK) and were then treated with DGG-100629 (2 μM) for 9 h. **b** Western blot analysis of cells treated with DGG-100629 for 6 h. **c** Fractionation assay in cells treated with DGG-100629 for 6 h. **d** ChIP assay in cells treated with DGG-100629 (2 μM) for 9 h. **e**, **f** HeLa cells were transfected with GFP-NFATc1 for 24 h and were then treated with DGG-100629 (2 μM) for 6 h (**e**) or 24 h (**f**). **e** Assessment of GFP-NFATc1 localization by visualization of green fluorescence with the IncuCyte system. Scale bar, 20 μm. **f** DDIAS expression in cells overexpressing GFP-NFATc1.

Since DGG-100629 was by far the most potent inhibitor of cancer cell growth among the three identified compounds, it was selected for further experiments. We previously demonstrated that binding of NFATc1 to a region of the DDIAS promoter located 350 bp upstream of the transcription start site is crucial for the regulation of DDIAS transcription^6^. To investigate the molecular mechanism by which DGG-100629 represses DDIAS transcription, the role of the NFAT element (i.e., the NFATc1-binding site) in the DDIAS promoter was explored by luciferase reporter assays in the presence of DGG-100629. DGG-100629 clearly inhibited the luciferase activity of both the P3 (−1205 base pair) and P5 (−350 base pair) DDIAS promoter fragments (Fig. 2a), indicating that the NFAT element plays a role in the DGG-100629-induced suppression of DDIAS transcription (Supplementary Fig. 2).

The role of NFAT signaling in DGG-100629-induced DDIAS suppression was further examined. DGG-100629 did not change the protein levels of NFATc1, NFATc2, NFATc3 or NFATc4 (Fig. 2b). A subcellular fractionation assay showed that DGG-100629 promoted the relocalization of NFATc1 from the nucleus to the cytoplasm (Fig. 2c). Moreover, a chromatin immunoprecipitation (ChIP) assay revealed that endogenous NFATc1 was recruited to the proximal DDIAS promoter region containing the NFAT binding sites and that DGG-100629 inhibited this recruitment (Fig. 2d). As expected, DGG-100629 suppressed the nuclear translocation of exogenous NFATc1 (Fig. 2e). However, NFATc1 overexpression slightly reversed the reduction in DDIAS expression (Fig. 2f). Collectively, these findings indicated that DGG-100629 suppressed DDIAS transcription by preventing NFATc1 translocation to the nucleus and, therefore, its recruitment to the DDIAS promoter.

### DGG-100629 inhibited the NFATc1/DDIAS pathway through the activation of JNK

**Fig. 3:**
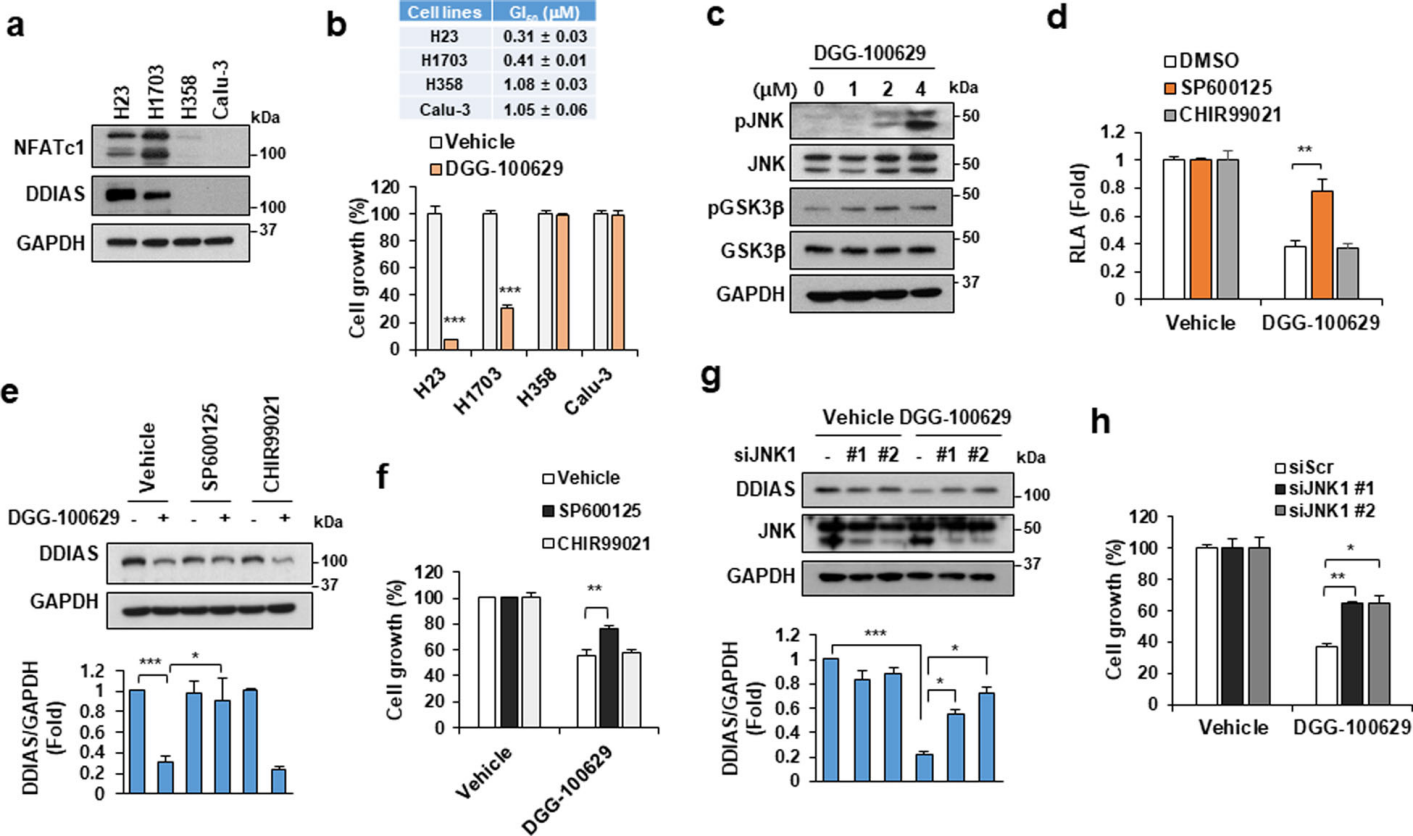
DGG-100629 activated JNK. **a** Expression of NFATc1 and DDIAS in NSCLC cells. **b** Cell growth assay in cells treated with DGG-100629 (0.5 μM). **c** Activation of JNK and GSK3β in H1703 cells treated with DGG-100629 for 6 h. **d**–**f** H1703 cells were treated with SP600125 (5 μM) and CHIR99021 (5 μM) in the presence of DGG-100629 (2 μM), and luciferase assays (**d**), western blotting with quantitative analysis (**e**), and cell growth assays (**f**) were performed. **g** Western blotting with quantitative analysis and **h** cell growth assay in H1703 cells transfected with siJNK1 for 24 h and then treated with DGG-100629 (2 μM) for 24 h. The data were obtained from triplicate experiments. The values shown are the mean±SEM values. *p < 0.05, **p < 0.01, ***p < 0.001.

Since a role of NFATc1 in the regulation of DDIAS expression in human lung cancers was previously demonstrated^6^, we explored the impact of DGG-100629 on NFATc1-regulated DDIAS expression in lung cancer cells. Western blot analysis showed that NFATc1 and DDIAS protein levels were higher in NCI-H23 (H23) and NCI-H1703 (H1703) cells than in NCI-H358 (H358) and Calu-3 cells (Fig. 3a). In addition, H23 and H1703 cells exhibited increased sensitivity to DGG-100629 (3.2-fold lower GI50) compared with H358 and Calu-3 cells (Fig. 3b). Nuclear export of NFATc1 is followed by its phosphorylation by glycogen synthase kinase-3β (GSK3β) or c-Jun NH(2)-terminal kinase (JNK) and by suppression of its transcriptional activity^22,23^. Notably, DGG-100629 was found to induce the activation of both GSK3β and JNK (Fig. 3c). Moreover, SP600125 (a JNK inhibitor) but not CHIR99021 (a GSK3β inhibitor) restored cell death and suppressed DDIAS promoter activity in the presence of DGG-100629 (Fig. 3d–f). Furthermore, *JNK1* silencing by a specific siRNA reversed DGG-100629-induced DDIAS downregulation and restored cell death (Fig. 3g, h). These results suggested that DGG-100629 suppressed DDIAS expression and cell growth by regulating JNK/NFATc1 signaling in NSCLC.

### DDIAS downregulation was involved in DGG-100629-induced apoptosis

**Fig. 4:**
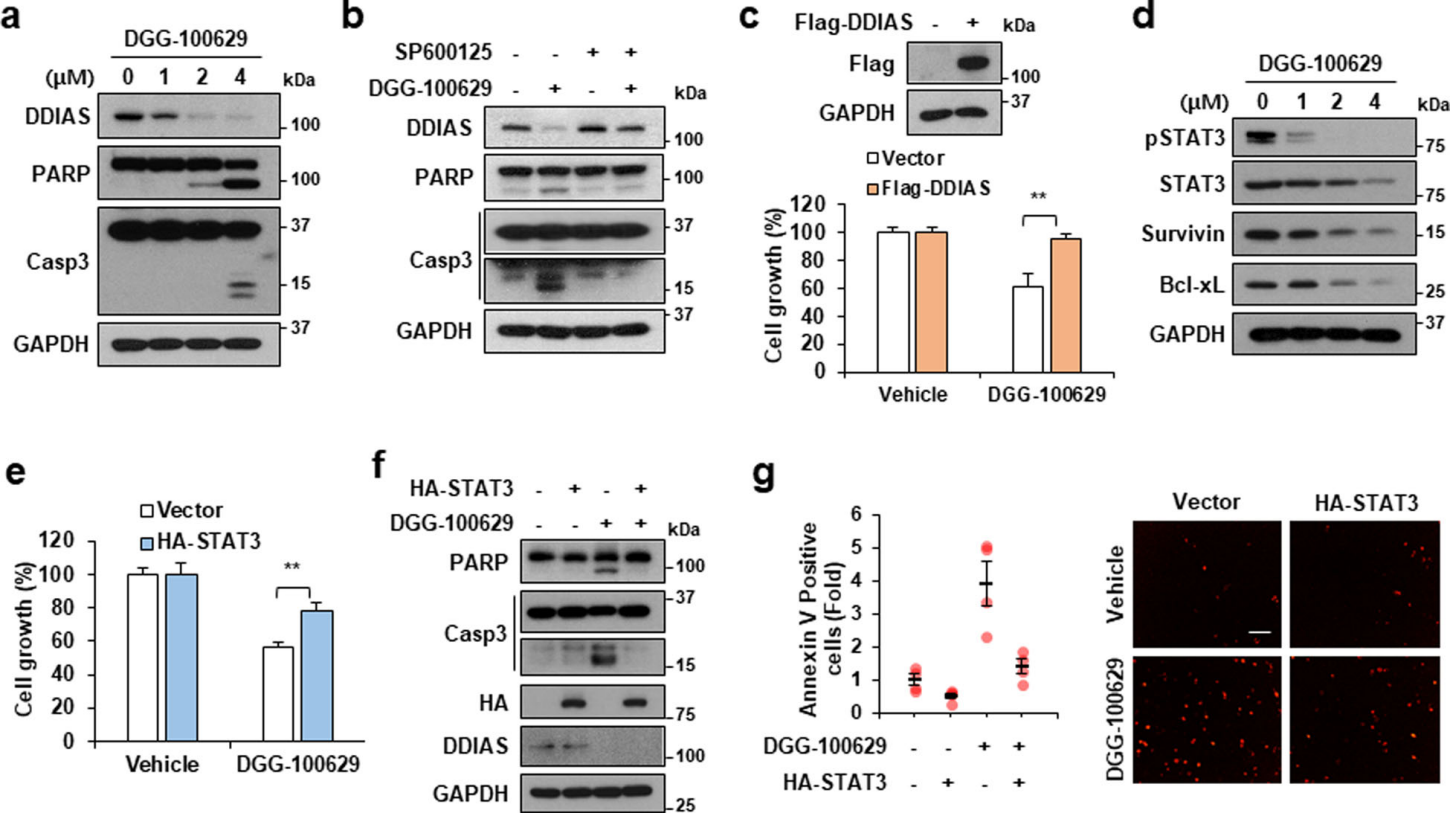
DDIAS and STAT3 overexpression prevented DGG-100629-induced lung cancer cell death. **a** Western blot analysis of H1703 cells treated with DGG-100629 for 24 h. **b** Western blot analysis of cells treated with SP600125 (5 μM) and DGG-100629 (2 μM). **c** Western blot analysis and growth of H1703 cells transfected with Flag-DDIAS and treated with DGG-100629 (2 μM). **d** Western blot analysis of cells treated with DGG-100629 for 24 h. **e**, **f** STAT3 overexpression after HA-STAT3 transfection prevented the apoptosis induced by DGG-100629 (2 μM). **g** Annexin V staining with the IncuCyte system. The scale bars represent 100 μm. The data were obtained from triplicate experiments. The values shown are the mean±SEM values. *p < 0.05, **p < 0.01.

We examined whether DGG-100629 caused apoptotic changes in lung cancer cells. DGG-100629 induced the cleavage of PARP and caspase-3, which was accompanied by a decrease in the DDIAS protein level (Fig. 4a). However, SP600125 blocked DGG-100629-induced cleavage of PARP and caspase-3 (Fig. 4b). Moreover, overexpression of Flag-DDIAS abrogated the inhibitory effect of DGG-100629 on cancer cell growth (Fig. 4c).

We previously reported that DDIAS promotes STAT3 tyrosine phosphorylation by inhibiting PTPRM and that DDIAS expression correlates with STAT3 phosphorylation in human lung cancer^11^. In the current study, we confirmed that DGG-100629 treatment decreased STAT3 phosphorylation, as well as the expression of the STAT3 targets Bcl-xL and survivin (Fig. 4d). In addition, HA-STAT3 overexpression attenuated DGG-100629-induced cell death, as well as PARP and caspase-3 activation (Fig. 4e, f). Moreover, STAT3 overexpression rescued cells from DGG-100629-induced cell death, as shown by annexin V staining (Fig. 4g). These results suggested that DGG-100629-induced DDIAS downregulation was required for the suppression of STAT3 activation and the promotion of apoptosis in NSCLC cells.

### DGG-100629 inhibited tumor growth in a mouse xenograft model

**Fig. 5:**
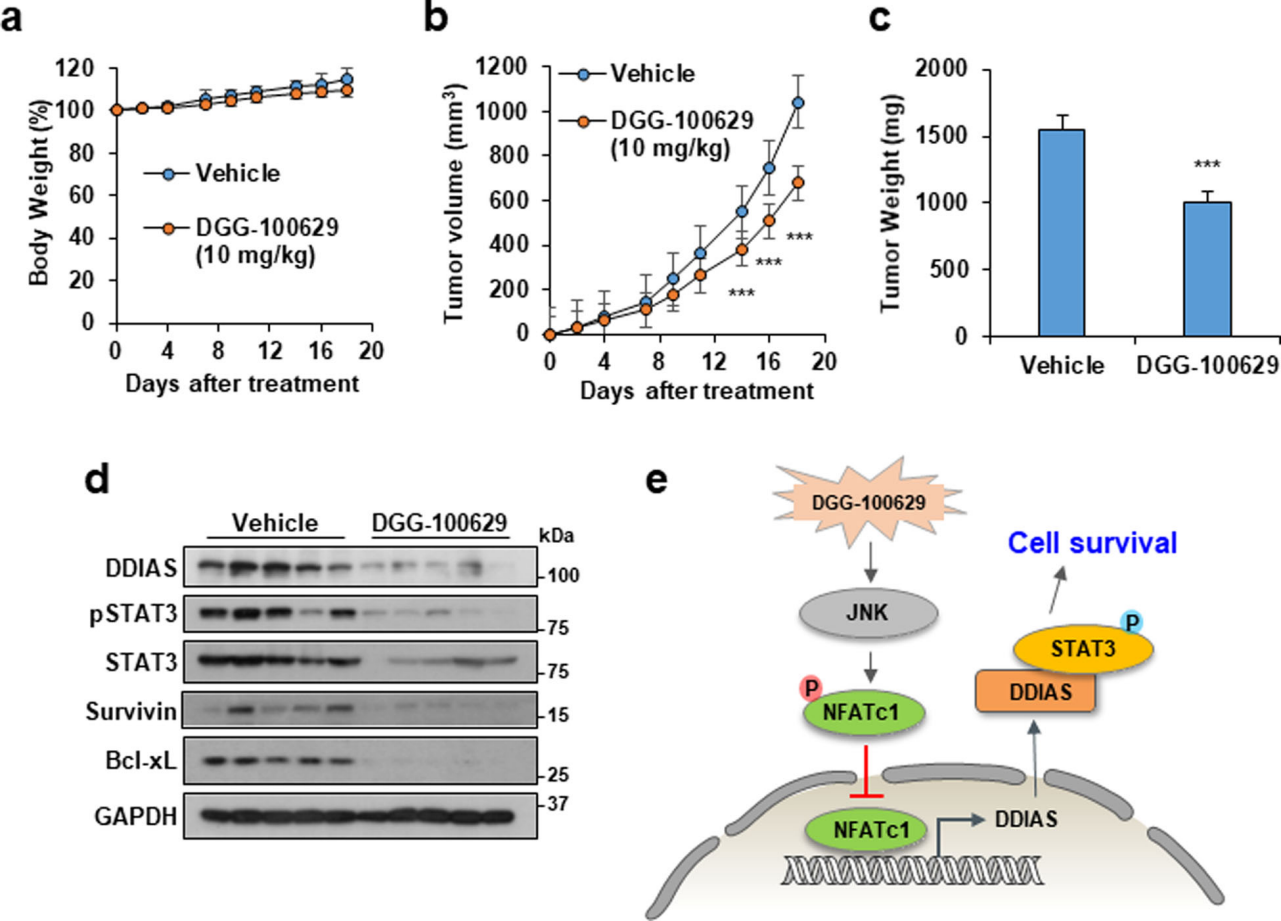
DGG-100629 suppressed tumor growth in vivo. **a**–**c** Body weight and tumor growth inhibition in the mouse xenograft assay. NCI-H1703 cells (9 × 10^6^ cells/mouse) were injected into BALB/c nude mice. DGG-100629 (10 mg/kg) was injected into tumors five times at 2-day intervals after the tumors reached an average volume of 77 mm^3^. Tumor volume data are shown (*n* = 5 mice/group; *p < 0.05, **p < 0.01). **d** DGG-100629 reduced the protein level of STAT3 and its targets in vivo, as determined by Western blotting. **e** Proposed model for the anticancer activity of DGG-100629.

To investigate the effect of DGG-100629 on tumor growth, a xenograft assay using H1703 cells was carried out. DGG-100629 (10 mg/kg) was administered to mice by intraperitoneal injection. DGG-100629-treated mice did not show significant changes in body weight (Fig. 5a), while they exhibited reductions of 34.8% and 35.3% in tumor volume and weight, respectively (Fig. 5b, c). Western blot analysis of resected tumors revealed that the protein levels of DDIAS and the STAT3 targets survivin and Bcl-2 were dramatically decreased in DGG-100629-treated tumors compared to vehicle-treated tumors (Fig. 5d). An in vivo xenograft assay demonstrated that the anticancer activity of DGG-100629 was associated with reduced STAT3 phosphorylation and downregulated expression of STAT3 target genes. Moreover, JNK activation by DGG-100629 inhibited the nuclear translocation of NFATc1, which resulted in DDIAS downregulation as well as inactivation of the STAT3 signaling pathway (Fig. 5e).

### DGG-100629 inhibited the growth of gefitinib-resistant lung cancer

**Fig. 6:**
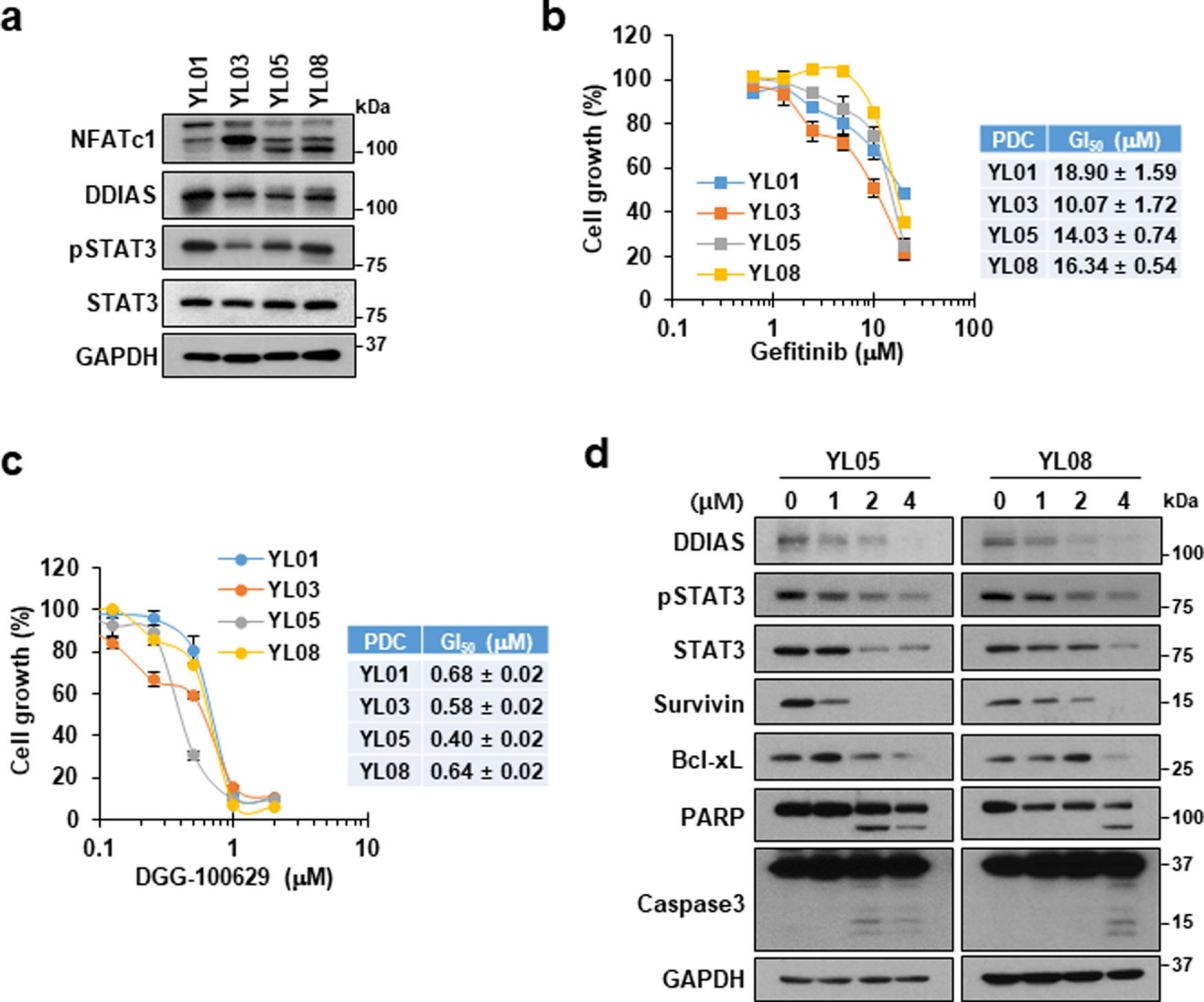
DGG-100629 inhibited the growth of gefitinib-resistant patient-derived lung cancer cells. **a**, **b** Cell growth inhibition by gefitinib (**a**) and DGG-100629 (**b**). Four PDC cell lines (YL01, YL03, YL05, and YL08) were treated with the compounds for 72 h. **c** Levels of NFATc1, DDIAS, pSTAT3, and STAT3, as determined by immunoblotting with anti-NFATc1, anti-DDIAS, anti-pSTAT3 (Y705), anti-STAT3, and anti-GAPDH antibodies. **d** Western blot analysis of YL05 and YL08 cells treated with DGG-100629 for 24 h.

Among NSCLC cell lines, H358 and Calu-3 cells are sensitive to gefitinib, while H23, H1299, and H1703 cells are resistant to gefitinib^24^. We observed that H23 and H1703 cells, which express high levels of DDIAS, were sensitive to DGG-100629. Therefore, patient-derived lung cancer cells (PDCs) were used to verify whether DGG-100629 could be a suitable therapeutic option for patients with gefitinib-resistant lung cancer. Therefore, the effect of DGG-100629 on the growth of various lines of gefitinib-resistant PDCs (YL01, YL03, YL05, and YL08) was examined. PDCs exhibited high levels of NFATc1, DDIAS, and activated STAT3 (Fig. 6a). Consistent with a previous report^19^, all tested PDC lines showed resistance to gefitinib, as indicated by a GI50 > 10 μM (Fig. 6b). However, DGG-100629 suppressed PDC growth at low micromolar concentrations (Fig. 6b, c). In addition, DGG-100629 inhibited the expression of DDIAS and STAT3 targets and promoted the death of YL05 (EGFR exon19del) and YL08 (wild-type EGFR, ALK mutation-positive) cells (Fig. 6d). These results suggested that NFATc1 and DDIAS in cancer cells contribute to high STAT3 levels and resistance to gefitinib and that DGG-100629 induces PDC apoptosis by downregulating DDIAS expression and suppressing STAT3 signaling.

## Discussion

Here, we identified DGG-100629 as a drug for the treatment of lung cancer via its suppression of DDIAS expression and inactivation of STAT3 signaling. DGG-100629 activated JNK, thereby preventing NFATc1 translocation to the nucleus and inhibiting the expression of DDIAS and STAT3 target genes, such as Bcl-xL and survivin. DGG-100629 displayed effective anticancer activity, both in vitro and in vivo, in lung cancer cells expressing high levels of NFATc1 and DDIAS and exhibiting gefitinib resistance.

We previously reported that NFATc1 but not NFATc2-4 is involved in DDIAS transcription^6^. Using siRNAs against NFATc1-4, we showed that NFATc1 regulates the promoter activity and mRNA level of DDIAS. In addition, NFATc1 but not NFATc2 is recruited to the promoter region of DDIAS, and its overexpression increases DDIAS expression. NFATc1 is an oncogene implicated in many cancers^25,26^. NFATc1 is highly expressed in lung cancer and promotes lung cancer cell survival and epithelial-mesenchymal transition^6,27^. Moreover, by promoting the expression of multiple target genes, including DDIAS, cyclin D1, IL-2, and IL-4, NFATc1 protects lung cancer cells against anticancer drug-induced death^6,28^. The transcriptional activity of NFATc1 is regulated by phosphorylation, which influences its nucleocytoplasmic distribution. NFATc1 activity is regulated by a variety of kinases. PIM1 promotes NFATc1 activity and stability^29,30^, while DYRK1a, GSK3β, and JNK inhibit NFATc1 transcriptional activity^22,23,31^. The present study demonstrated that DGG-100629 activated JNK, which in turn induced NFATc1 phosphorylation and cytoplasmic translocation, ultimately suppressing DDIAS transcription. Notably, GFP-NFATc1 overexpression did not significantly alter the effect of DGG-100629 on DDIAS expression (Fig. 2f), implying that the intracellular localization of NFATc1, rather than its protein level, is crucial for DDIAS transcription.

Quinoxaline derivatives have been reported to activate the JNK and p38 MAPK pathways, resulting in cancer cell death^32–34^. Consistent with previous findings, we found that DGG-100629 induced JNK phosphorylation and that both transcriptional and pharmacological suppression of JNK reversed DGG-100629-induced DDIAS downregulation and restored cancer cell growth (Fig. 3). Notably, a previous study demonstrated that quinoxaline derivatives of DGG-100629 inhibit the Wnt/β-catenin pathway, leading to a decrease in the β-catenin protein level^18^, which is believed to be associated with the phenotype of DDIAS knockdown^9^. Even though the lead compound has the same core skeleton of as previous research targets, the lead compound with the most potent targeted activity toward DDIAS transcription, DGG-100629, has a building block chemical structure (2-ethoxy ethyl amine group) quite different from that of the previous targets in NSCLC or the Wnt2/β-catenin pathway (2-ethoxy amine group). The piperazine side chain at the 2-position is much longer than that of the previous research targets. On the other hand, additional effects of DGG-100629 on cell survival may not be excluded. We assumed that the relatively poor efficacy of DGG-100629 in vivo may be due to its low microsomal stability and poor pharmacokinetic properties (Supplementary Fig. 3). Further structural modifications may be needed to improve the pharmacokinetic profile and in vivo efficacy of DGG-100629.

High expression of NFATc1 and DDIAS was previously found to be associated with drug resistance in lung cancer^6,28^. Interestingly, we found that the expression of NFATc1 and DDIAS was elevated in H23 and H1703 cells, as well as in four gefitinib-resistant PDCs, but was downregulated in gefitinib-sensitive lung cancer cells, such as H358 and Calu-3 cells (Figs. 3a; 6a, c; and Supplementary Fig. 4). DGG-100629 markedly inhibited the proliferation of gefitinib-resistant PDCs, as well as H23 and H1703 cells. Our data may suggest a novel strategy for the treatment of gefitinib-resistant lung cancers with high expression of NFATc1 and DDIAS.

Elevated DDIAS expression promotes STAT3 activity^11^. In human lung cancer cells and tissues, DDIAS expression is strongly correlated with the level of phospho-STAT3 Y705, which supports the role of STAT3 phosphorylation in lung tumorigenesis^11^. DGG-100629 reduced the levels of total and phosphorylated STAT3 but not its mRNA level (Fig. 4d and Supplementary Fig. 5). Since DGG-100629 caused DDIAS downregulation, the impact of DDIAS knockdown on STAT3 protein expression and phosphorylation was examined. In fact, DDIAS knockdown by two different siRNAs reduced the STAT3 protein level in both H23 and H1703 cells (Supplementary Fig. 6). The DDIAS protein level was previously found to correlate with both the total and phosphorylated STAT3 (phospho-STAT3 Y705) levels in lung cancer cell lines^11^. However, *DDIAS* knockdown suppressed STAT3 Y705 phosphorylation but did not affect the total protein level of STAT3 in the presence of IL-6 stimulation. The level of STAT3 might be regulated by different mechanisms depending on the cellular context. STAT3 was reported to be regulated by posttranslational modifications such as phosphorylation, acetylation, oxidation, and ubiquitination^35–38^. PDLIM2, Fbw7, and constitutive photomorphogenic 1 (COP1) were identified as E3 ligases for STAT3 in T helper 17 cells, diffuse large B-cell lymphoma (DLBCL) cells, and prostate cancer cells, and several proteins, including TMF/ARA160, are involved in proteasomal STAT3 degradation^39–43^. In addition, TRAF6 mediates K63-linked polyubiquitination of STAT3, thus repressing its transcriptional activity, independent of STAT3 degradation^37^. Monoubiquitination of STAT3 at K97 enhances the expression of antiapoptotic genes by promoting the formation of the bromodomain-containing protein 4 (BRD4) complex^44^. Further studies are needed to confirm the role of DDIAS in the regulation of STAT3 protein stability and in the ubiquitin-proteasome system.

We suggest that DDIAS-targeting agents may be suitable for the personalized treatment of patients with high DDIAS/STAT3 expression. Our data provide mechanistic insights into the tumorigenic effects of DDIAS, linking NFATc1 function in immune cells to the role of STAT3 in the proliferation and survival of lung cancer cells. Finally, our findings suggest an alternative strategy for the management of gefitinib-resistant lung cancers with high DDIAS expression.

## Supplementary information

supplementary materials
