# Supplementary material for: DGG-100629 inhibits lung cancer growth by suppressing the NFATc1/DDIAS/STAT3 pathway
Source: Exp Mol Med. 2021 Apr 15;53(4):643–53. doi: 10.1038/s12276-021-00601-2 (PMC8102629; doi:10.1038/s12276-021-00601-2)
Supplement: Supplementary file 1 — supplementary materials [file 12276_2021_601_MOESM1_ESM.pdf]

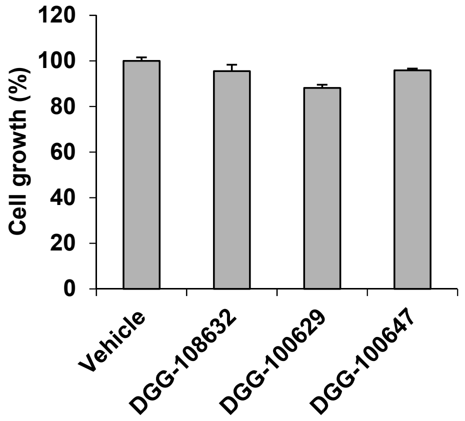


**Supplementary Fig. S1** The effect of DGG-108632, DGG-100629, and DGG-100647 on the growth inhibition of WI-38 at 5mM for 72 h.

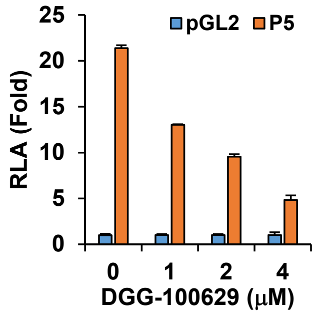


**Supplementary Fig. S2** HeLa cells were co-transfected with reporter plasmids containing the luciferase gene under the control of the *DDIAS* promoter (P5 or pGL2) and TK-Ren, and then treated with DGG-100629 for 9 h.

**a**

| Liver microsomes | Cofactor | % remaining after 30 min incubation | |
|---|---|---|---|
| | | DGG-100629 | Buspirone* |
| Human | +NADPH | 24.84 ± 0.37 | 2.93 ± 0.05 |
| | -NADPH | 93.54 ± 1.61 | 93.11 ± 0.43 |
| Mouse | +NADPH | 2.43 ± 0.32 | 0.19 ± 0.02 |
| | -NADPH | 94.94 ± 0.60 | 94.37 ± 1.33 |

**b**

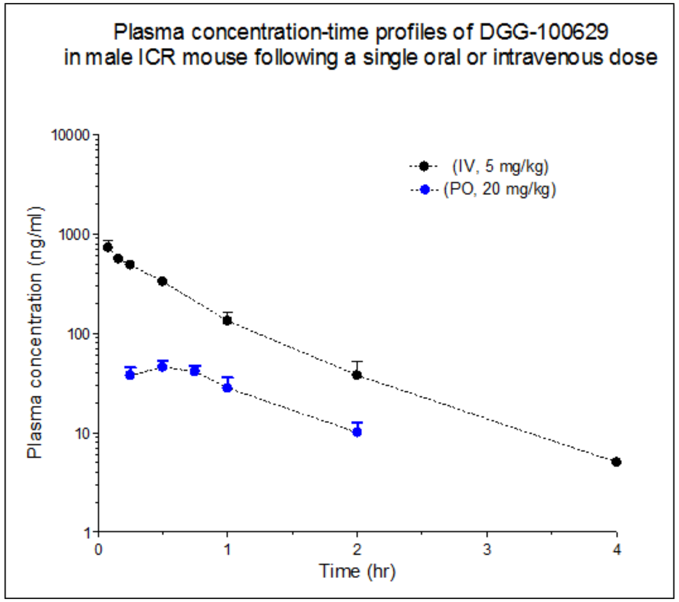


**Supplementary Fig. S3** Microsomal stability and Pharmacokinetics assay of DGG-100629. (a) Microsomal stability was determined in human or mouse liver microsomes (0.5 mg protein/ml) at 37 C for 30 min an the absence and presence of NADPH (1mM) using 1 mM test assay. (b) Pharmacokinetics assay. In vitro and in vivo. Plasma concentration-time profiles of DGG-100629 following a single dose in male ICR mice. A single dose of ICR mice was given intravenously (5 mg/kg; n=3, ●) or orally (20 mg/kg; n=3, ●). Each point represents mean ± S.D. (n=3). The plasma concentration of DGG-100629 was determined for 24 hr post-dosing. It was not detected in the plasma 4hr or 2h after the intravenous or oral administrations.

| Cell Lines | Gefitinib $GI_{50}$ (μM) |
|---|---|
| H23 | 53.0 ± 4.2 |
| H1703 | 16.2 ± 1.0 |
| H358 | 2.8 ± 0.4 |
| Calu-3 | 2.0 ± 0.6 |

**Supplementary Fig. S4** Gefitinib sensitivity in lung cancer cells. H23, H1703, H358, and Calu-3 cells were treated with gefitinib for 72 h, and cell growth was evaluated by an sulforhodamine B (SRB) assay.

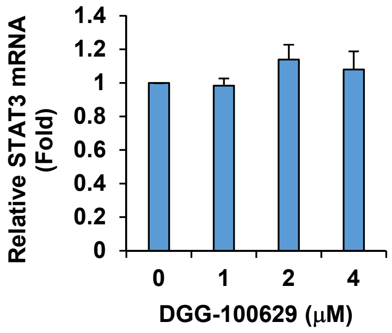


**Supplementary Fig. S5.** RT-PCR analysis of STAT3 mRNA expression in H1703 cells treated with DGG-100629 for 24 h. GAPDH was used as an internal control.

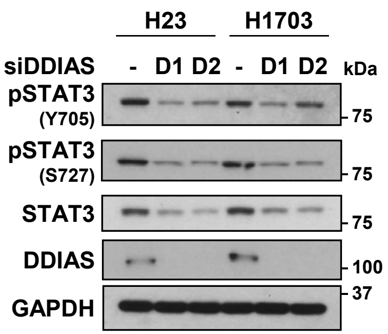


**Supplementary Fig. S6** Western blot analysis of STAT3 protein expression in H23 and H1703 cells knocked down for DDIAS via transfection with specific siRNAs (40 nM) for 72 h.
